# The Cost of Drugs

**Published:** 1905-09-30

**Authors:** 


### HOSPITAL ADMINISTRATION.

### CONSTRUCTION AND ECONOMICS.

### THE COST OF DRUGS.

A CONTRIBUTION TO HOSPITAL ECONOMICS.

By the Chief Dispenser of a Large Hospital.

In recent months the spirit of inquiry into the affairs of large hospitals has been abundantly manifested in the current literature of magazines and daily papers. The results have shown that it was not wholly uncalled for. Some points, indeed, are still requiring elucidation, and not the least important of these is the broad question whether it is economically sound policy for such institutions to prepare their own pharmaceutical preparations or galenicals, or to buy them ready made in the open market. Like most other questions it does not admit of a simple answer on rule-of-three lines. Moreover, the question has generated not a little heat in certain quarters, but in an inquiry of this kind there is, happily, no need for personal or even special reference to any particular institution, as the truth of the matter may be ascertained by reference to a hypothetical case.

For purposes of argument let it be supposed that the following remarks are applicable to a general hospital of 600 beds, in London or the provinces, for nowadays it really matters little where, and let it be further clearly apprehended that making preparations refers to pharmaceutical preparations and analogous supplies—such as aerated waters, etc., and in no case does it mean fine chemicals.

Bearing these points in mind, let us look briefly at some of the considerations that suggest themselves for or against. In favour of purchasing all such supplies and simply confining the duty of the pharmacist to compounding we have adduced arguments such as the following :—

1. Supplies (*i.e.* finished pharmaceutical preparations, such as compound and concentrated infusions, tinctures, liquid extracts, etc.), being bought in the open market by competitive contract are obtained at the cheapest rate.
2. The wages bill is reduced.
3. The materials in question cannot be produced pound for pound as cheaply as in a wholesale warehouse, which is, of course, largely begging the question.

A few observations of the nature of a corollary may be made here. An institution under the jurisdiction of a board or superintendent taking these propositions for guidance will, of course, have no occasion to sanction the purchase of apparatus more than is absolutely necessary for compounding—that is to say, in the opinion of those who advocate making, the laboratory of such an institution is poorly equipped. Not to add any further detail, this is practically the case for restricting the work to compounding.

Now let us turn to some considerations which suggest themselves on looking further into these arguments. By the purchase of supplies in the open market we no doubt buy at the cheapest rate; but the same observation applies to the raw materials, and in their case the purchaser sees what he is getting, whereas, with most pharmaceutical products or galenicals there is unlimited scope for the cultivation of the virtue of honesty. Cheapness has inextricably associated with it the idea of quality. Indeed, it is with this factor that the suggestion of cheapness has to make its defence. A product which no analyst could describe as not responding to official characters and tests may be concocted from very indifferent materials. Liquid extract of cascara may be made from bark costing less than 35s. per cwt., or from a thoroughly-matured bark costing possibly three times that sum; tincture of digitalis—a very important preparation—may be made from the veriest rubbish, and the finished product look as well as that which has been obtained from the finest leaves; the same remark applies to tincture of hyoscyamus, and with still greater force to tincture of strophanthus, a preparation which, when carefully made by Professor Fraser's method from the finest materials and with skilful attention to details, is of the highest value. But the same observations ramify in all directions of pharmacy. There are half-a-dozen grades of rhubarb, all making products which are ocularly, and for that matter analytically, correct, and yet are of widely different therapeutic value. Rice starch may be quoted at anything from 20s. to 35s.; sweet spirit of nitre, a product still extensively used, may be purchased to-day responding to the requirements of the British Pharmacopœia, and to-morrow it does not; colocynth is another substance appearing in different grades, most of which will give preparations whose impurities require considerable skill for their detection. But it is unnecessary to multiply illustrations, the fact is simply a commonplace. So that in the purchase of drugs cheapness is eminently a relative term, and in this work more than any other suggests the necessity for the greatest circumspection. But it may be replied, that purchases are made under guarantee of good quality and from firms of repute—is this not a safeguard?

To answer this let us see how purchasing such preparations works out in practice, always retaining the appreciation of quality as a *sine qua non.* A number of samples are before you, all equally "guaranteed," and other things being equal you take the lowest. This leads to an endeavour on the part of one or other of the unsuccessful offerers to cut in next time, and the gradation process sets in, and imperceptibly you are pushed into buying what is fundamentally an inferior article. In the purchase of medicine it is the easiest process in the world, and a real difficulty with all who have to buy such materials. Practically it comes eventually to two alternatives—either you take the lowest "guaranteed" offer with the result I have indicated, or deciding to be secure in quality, you buy from a house of good standing, and eliminating the competitive element, pay them what is mutually considered a fair price—which is paying for security in quality. But I shall return to the question of "guarantee" later. Meantime we may conveniently turn to a consideration of the second argument—the wages bill. It is presumed if you make these things, a staff must be maintained for the purpose, or at least you need more employés than would be otherwise required. If it were necessary to keep an expensive staff for the purpose of making and testing pharmaceutical preparations the argument might have considerable weight, especially if we agreed to put aside for a moment references to quality. Now this involves two points of importance, management and equipment. Under methodical and proper supervision no such staff is required. Pharmacists who have been trained in a good-class country business, where most of the pharmaceutical work is done, will serve the purpose perfectly. Here a survey of the actual practical conditions may be introduced. In the laboratory of a large institution of the kind under contemplation, on two or even three days each week—Monday, possibly Wednesday, and again on Saturday, the staff will be almost wholly occupied with actual compounding; these are periods of accumulation and discharge, as it were, in the wards. But, on the other three working days, there may not be anything like the same pressure, and the work, such as it is, can be made to fill in the spare time. Again, here as elsewhere there are seasonal oscillations in the work; there is more to be done at one period of the year than another. Now there are two propositions perfectly clear from the above. (*a*) The whole staff was necessary to do the routine work. It is not in this work, as in many other situations, what cannot be done to-day, will be done to-morrow. The medicines, etc., are wanted and have to be supplied practically *instanter*. This is a simple practical fact. (*b*) There are necessarily periods when times of less pressure could have their full working value extracted by attention to other things as well, such as the preparation of galenicals. Again, as to equipment, the other factor in efficient and economical working, this is an exceedingly important component in such an estimate as we are attempting. This is not to be understood as a costly thing. It is a matter of £150 at most over the cost of what must be considered ordinary working appliances. These appliances enable the work to be expeditiously and economically done.

(*To be continued.*)

